# Importance of Preoperative Left Ventricle Volume Assessment in Asymptomatic Tetralogy of Fallot Infants

**DOI:** 10.5761/atcs.oa.25-00077

**Published:** 2025-11-26

**Authors:** Akinori Hirano, Takaya Hoashi, Shigeki Yoshiba, Ryusuke Hosoda, Yuji Fuchigami, Yukino Iijima, Takaaki Suzuki

**Affiliations:** 1 Department of Pediatric Cardiac Surgery, Saitama Medical University International Medical Center, Hidaka, Saitama, Japan; 2 Department of Pediatric Cardiology, Saitama Medical University International Medical Center, Hidaka, Saitama, Japan

**Keywords:** tetralogy of Fallot, primary repair, left ventricular volume, echocardiography

## Abstract

**Purpose:**

The study investigated the importance of left ventricular volume assessment before primary repair in asymptomatic tetralogy of Fallot (TOF) patients.

**Methods:**

Forty-two asymptomatic TOF patients who underwent preoperative cardiac catheterization at a median age of 4.7 months interquartile range [IQR], 4.0–5.3) between 2013 and 2023 were enrolled. Asymptomatic TOF was defined as room air oxygen saturation ≥85% without duct-dependent circulation. Left ventricular end-diastolic volume (LVEDV) as a percentage of predicted normal (LVEDV%N) was measured using the single-plane area–length method. Correlation with echocardiographic parameters was assessed.

**Results:**

The median LVEDV%N was 107% (IQR, 87.5–139.5). Five patients (11.6%) had LVEDV%N ≤80%. One patient with the lowest LVEDV%N (62%) underwent a modified Blalock–Taussig shunt instead of primary repair. The remaining four patients had a small pulmonary valve annulus (PVA) (Z-score −4.2 to −6.6) and underwent transannular patch repair. Seven additional patients underwent transannular patch repair due to total conal ventricular septal defect (n = 5) or patent ductus arteriosus with small PVA (n = 2). LVEDV%N showed a weak correlation with 1-month echocardiographic parameters (R^2^ = 0.27–0.347).

**Conclusions:**

Left ventricular volume assessment prior to primary repair is essential in asymptomatic TOF patients. There were cases with small LV for whom primary repair was deferred, or whose PVA was also small; therefore, transannular patch repair was selected.

## Abbreviations

IQR
: interquartile range
LV
: left ventricle
LVEDd
: left ventricular end-diastolic diameter
LVEDd%N
: left ventricular end-diastolic diameter as a percentage of predicted normal
LVEDV
: left ventricular end-diastolic volume
LVEDVI
: left ventricular end-diastolic volume index
LVEDV%N
: left ventricular end-diastolic volume as a percentage of predicted normal
MVD
: mitral valve diameter
MVD%N
: mitral valve diameter as a percentage of predicted normal
TOF
: tetralogy of Fallot

## Introduction

In tetralogy of Fallot (TOF), insufficient pulmonary blood flow leads to underdevelopment of both the branch pulmonary arteries (PAs) and the left ventricle (LV). The American Association for Thoracic Surgery (AATS) expert consensus in 2022 recommended palliative interventions for TOF patients with significant hypoxia or a history of anoxic spells, while primary repair at 3–5 months of age is recommended in asymptomatic patients.^1)^ However, it remains unclear whether all asymptomatic TOF patients have adequate left ventricular volume for primary repair without preliminary palliation. Previous studies have demonstrated that total correction is safest in patients with left ventricular end-diastolic volume (LVEDV) greater than 70% of predicted normal (LVEDV%N), though it may be feasible with an LVEDV%N as low as 60%, corresponding to an LVEDV index (LVEDVI) of 30 mL/m^2^.^2–5)^

Whereas neonatal primary repair should be avoided due to its high mortality,^6–8)^ the operative or interstage mortality rate after palliative shunt operation during the neonatal and early infantile period is not low at all.^1)^ Hence, if prediction of LV volume just before primary repair at 3–5 months of age was possible during the early infantile period, unnecessary palliative operation can be avoided.

In addition to promoting LV or PA growth, palliative shunt has been reported to contribute to the growth of the pulmonary valve (PV) annulus, which would facilitate valve-sparing repair instead of transannular patch (TAP) repair.^9–11)^ Therefore, even though patients are free from any symptoms and have adequate LV volume to undergo primary repair, a preceding shunt operation will be justified if their TOF is repaired by the TAP procedure due to a small PV annulus, to prevent harmful later pulmonary regurgitation.

This study reviewed LVEDV%N prior to primary repair and its relation to postoperative outcomes in asymptomatic TOF patients. We also examined transthoracic echocardiography (TTE) parameters measured at around one month of age as potential predictors of preoperative LV volume.

## Patients and Methods

### Study design and patient selection

Asymptomatic TOF was defined as room air oxygen saturation (SpO_2_) ≥85% measured by pulse oximetry on the right hand in quiet, awake patients with stable readings for ≥5 minutes, combined with the absence of feeding difficulties, appropriate weight gain, no anoxic spells, and normal activity tolerance.^12,13)^ Patients with duct-dependent pulmonary circulation were excluded. Between January 2013 and December 2023, 55 consecutive TOF patients underwent surgical intervention at our center, of whom 43 asymptomatic patients were enrolled as candidates for primary total correction. One patient requiring home oxygen therapy was subsequently excluded from analysis as this represented symptomatic status, leaving 42 patients for final analysis. The median age and weight at preoperative catheterization were 4.7 months (interquartile range [IQR], 4.0–5.3) and 6.0 kg (IQR, 5.3–6.9), respectively. Initial TTE was performed at a median age of 31 days (IQR, 24–45) as a baseline evaluation.

In our institution, based on previous clinical experience, we have adopted a policy of performing primary repair in asymptomatic TOF patients with an LVEDV%N of 70% or more. This is based on Nomoto’s report,^4)^ which indicated that patients with an LVEDV%N of less than 70% required significantly higher catecholamine doses postoperatively and had unstable hemodynamics. Although some literature reports^2–5)^ suggest primary repair may be possible with an LVEDV%N as low as 60%, we have employed a more conservative criterion to ensure safer perioperative management.

### Patient background

Preoperative patient characteristics are summarized in **Table 1**. The study cohort comprised 29 males and 13 females, with comparable clinical features between genders. Chromosomal abnormalities were present in 6 patients (14.3%): 3 with trisomy 21 and 3 with 22q11.2 microdeletion syndrome. Baseline characteristics, preoperative left ventricular parameters, and surgical strategies were similar between patients with and without chromosomal abnormalities. Persistent left superior vena cava was present in 5 patients (11.9%), but its presence did not influence the choice of surgical strategy between primary repair and staged approach. Other associated cardiac anomalies were limited to minor anatomical variations that did not affect surgical planning or outcomes.

**Table 1:** Preoperative and operative characteristics of 42 asymptomatic tetralogy of Fallot patients undergoing primary repair

Variables	n (%) or median (IQR)
Number of patients	42
Female	13 (31.0%)
Gestational age (weeks)	37 (37–39)
Prematurity	1 (2.4%)
Chromosome anomaly	6 (14.3%)
Pulmonary valve anatomy	
Bicuspid	34 (81.0%)
Tricuspid	8 (19.0%)
Associated lesions (other than ventricular septal defect, atrial shunt, or pulmonary stenosis)	
Persistent left vena cava	5 (11.9%)
Right aortic arch	4 (9.5%)
Retroaortic innominate vein	2 (4.8%)
Single coronary artery	1 (2.4%)
Transthoracic echocardiogram around 1 month	
Age (days)	32 (24–46)
Persistent ductus arteriosus	5 (11.9%)
Left ventricular end-diastolic diameter (mm)	16.5 (14.9–18.7)
Left ventricular end-diastolic diameter (% of predicted normal)	84.5 (77.1–99.1)
Mitral valve diameter (mm)	11.0 (10.0–12.1)
Mitral valve diameter (% of predicted normal)	87.6 (81.7–91.6)
Preoperative SpO_2_ (room air, %)	94.4 (85–100)
Beta-blocker use	28 (66.7%)
Preoperative catheter examination	
Age (months)	4.7 (4.0–5.3)
Weight at catheter (kg)	6.0 (5.3–6.9)
Body surface area (m^2^)	0.30 (0.29–0.33)
Left ventricular end-diastolic volume (mL)	15.8 (13.7–18.7)
Left ventricular end-diastolic volume index (mL/m^2^)	52.6 (43.6–64.4)
Left ventricular end-diastolic volume (% of predicted normal)	107 (87.5–139.5)
Nakata index (pulmonary artery index) (mm^2^/m^2^)	299 (214–390)
TOF repair	
Age at operation (months)	6.3 (5.5–7.5)
Weight at operation	6.5 (5.6–7.4)
Procedure for right ventricular outflow tract	
Transannular patch	11 (26.2%)
Valve sparing	30 (71.4%)
Right ventriculotomy	34 (81.0%)
Cardiopulmonary bypass time (minutes)	177 (156–202)
Aortic cross clamp time (minutes)	121 (99–132)
Second bypass run	3 (7.1%)
Extracorporeal membrane oxygenation	0 (0%)

Categorical data are presented as number (%) and continuous data as median (IQR). IQR: interquartile range; SpO_2_: oxygen saturation; TOF: tetralogy of Fallot

Initial TTE revealed median left ventricular end-diastolic diameter and mitral valve diameter (MVD) of 84.5% (IQR, 77.1–99.1) and 87.6% (IQR, 81.7–91.6) of predicted normal values, respectively. Preoperative catheterization demonstrated a median LVEDV of 15.8 mL (IQR, 13.7–18.7), equivalent to 107% (IQR, 87.5–139.5) of predicted normal value, with a Nakata index of 299 mm^2^/m^2^ (IQR, 214–390).

### Study endpoints and measurements

The study evaluated the following 3 primary endpoints:

1. LVEDV%N assessed by preoperative cineangiography
2. Clinical course of patients with small LV
3. Correlation between early echocardiographic parameters and preoperative LVEDVi or Nakata index

Left ventricular volumes were measured using the single-plane area-length method with Medis Suite 4.0 software (Medis Medical Imaging Systems BV, Leiden, the Netherlands). The single-plane method was applied consistently due to software limitations precluding biplane analysis. The LVEDV%N was calculated based on normal left ventricular volume reference values for children according to body surface area, as reported by Nakazawa et al.^2)^ This method is widely accepted as a standard approach for left ventricular volume assessment in children and is commonly used for surgical decision-making in patients with congenital heart disease.

### Statistical analysis

All continuous variables are presented as medians (IQR), and categorical variables as numbers (%). Correlations among different variables were estimated using the Pearson’s correlation coefficient (r). For TAP patient comparisons between LVEDV groups, Mann–Whitney U test was applied due to the small sample size. Fisher’s exact test was used for categorical variables. For comparative analysis between LVEDV%N subgroups, the Mann–Whitney U test was applied due to small sample size. Continuous variables are presented as mean ± standard deviation. All p-values were 2-sided, and p-values <0.05 were considered statistically significant. Statistical analyses were performed using Excel for Microsoft 365 (Microsoft corporation, Redmond, Washington, USA).

## Results

### Overall outcomes

All patients had room air SpO_2_ ≥85% (range, 85%–100%; mean, 94.4% ± 4.1%), confirming asymptomatic status. Beta-blocker therapy was used in 28 patients (67.4%) for prophylactic management. Surgical repair was performed at a median age of 6.3 months (IQR, 5.5–7.5), with TAP reconstruction required in 11 patients (26.2%) (**Table 1**). Median cardiopulmonary bypass and aortic cross-clamp times were 177 minutes (IQR, 156–202) and 121 minutes (IQR, 99–132), respectively. Three patients (7.1%) required a second bypass run. At the completion of repair, the median right ventricular-to-systemic pressure ratio was 0.60 (IQR, 0.50–0.70). There were no operative deaths.

### Distribution of preoperative LVEDV

**Fig. 1:**
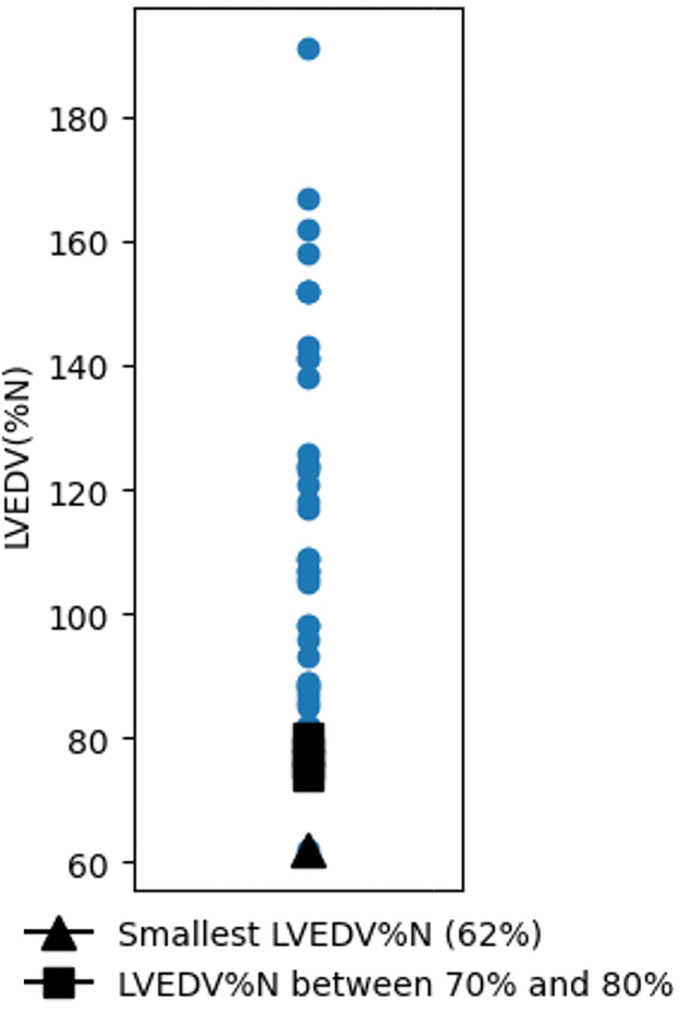
Distribution of preoperative left ventricular end-diastolic volume (LVEDV). A closed triangle denotes the patient with the smallest LVEDV% of predicted normal (LVEDV%N: 62%). Closed squares represent patients with an LVEDV%N between 70% and 80%.

The median LVEDV%N was 107% (range, 62%–191%; IQR, 88%–140%) (**Fig. 1**). One patient had LVEDV%N of <70%, and four patients had LVEDV%N between 70% and 80%.

### Clinical courses of small LV patients

In the patient with the smallest LV, catheterization revealed an LVEDV%N of 62% (LVEDVI: 35.5 mL/m^2^). Cardiac magnetic resonance imaging confirmed small LV volume (LVEDV%N: 73%; LVEDVI: 41.7 mL/m^2^). Despite the absence of hypoxic spells, we elected to perform a modified Blalock–Taussig shunt. Pre-intracardiac repair catheterization demonstrated increased LVEDV%N to 120%, which allowed valve-sparing repair with an uncomplicated postoperative course.

All four patients with an LVEDV%N between 70% and 80% required TAP reconstruction (**Table 2**). There were no early or late mortalities, but patient 1 (LVEDV%N 74%) exhibited significant left heart failure postoperatively, resulting in prolonged intensive care unit stay with high catecholamine requirements. Follow-up catheterization at one year revealed persistent diastolic dysfunction with elevated left ventricular end-diastolic pressure of 13 mmHg, right ventricular systolic pressure was almost 60% of LV systolic pressure, with no residual shunt. Patient 2 required reoperation for residual ventricular septal defect (VSD), and patients 3 and 4 recovered uneventfully (**Table 3**).

**Table 2:** Clinical courses of patients whose left ventricular end-diastolic volume percentage of predicted normal of 70%–80% (n = 4) (preoperative and surgical characteristics)

Patient	LVEDV%N (pre-ICR) (%)	MVD%N (pre-ICR) (%)	Surgical procedure	RVp/LVp ratio (post-ICR)	CVP (post-ICR) (mmHg)
1	74	89.8	TAP	68	14
2	76	69.5	TAP	55	11
3	78	75.1	TAP	79	13
4	80	78.1	TAP	75	9

LVEDV%N: left ventricular end-diastolic dimension % of predicted normal; ICR: intracardiac repair; MVD%N: mitral valve diameter % of predicted normal; RVp: right ventricular pressure; LVp: left ventricular pressure; CVP: central venous pressure; TAP: transannular patch

**Table 3:** Postoperative course and early outcomes of patients whose left ventricular end-diastolic volume percentage of predicted normal of 70%–80% (n = 4)

Patient	Ventilation (days)	ICU stay (days)	MCI	Hospital stay (days)	RVp/LVp ratio (1 POY) (%)	LVEDP (1 POY) (mmHg)	Early complications
1	8	23	13	32	59	13	Heart failure, PD
2	0	12	3	21	83	1	Re-intervention (residual VSD)
3	2	9	5	24	51	9	None
4	1	6	5	17	60	15	None

TAP: transannular patch; MCI: maximum catecholamine index; LVEDP: left ventricle end-diastolic pressure; PD: peritoneal dialysis; POY: post operative year; ICU: intensive care unit; LVp: left ventricular pressure; RVp: right ventricular pressure; VSD: ventricle septal defect

Among the 11 TAP patients, statistical comparison between those with an LVEDV%N <80% (n = 3) and ≥80% (n = 8) revealed no significant differences in anatomical characteristics. PV Z-scores were −5.27 ± 1.00 versus −4.18 ± 1.79 (p = 0.127), and PA index (PAI) were 298 ± 62 versus 365 ± 105 (p = 0.200, Mann–Whitney U test). All TAP patients had adequate PA development (PAI >150).

Detailed analysis of TAP indications revealed anatomical heterogeneity among the 11 cases (**Table 4**). Five patients had VSD with total conus defect requiring patch extension, two had small patent ductus arteriosus (PDA) with preserved systemic saturation but inadequate PV annulus. Among the remaining four patients with combined small LV volume and small PV annulus (including the three with an LVEDV%N of 70%–80% and one with an LVEDV%N exactly 80%).

**Table 4:** Details of patients who underwent transannular patch repair as the primary repair (n = 11)

No.	Reason for TAP	Preop. PV Z-score	Preop. LVEDV%N	Preop. Nakata index
1 (also No. 1 in **Tables 2** and **3**)	Small PV	−6.6	74	281
2 (also No. 2 in **Tables 2** and **3**)	Small PV	−4.2	76	381
3 (also No. 3 in **Tables 2** and **3**)	Small PV	−5.0	78	233
4 (also No. 4 in **Tables 2** and **3**)	Small PV	−5.3	80	158
5	Small PV, with PDA	−4.7	87	209
6	Small PV, with PDA	−5.2	141	272
7	Total conus defect type VSD	−2.3	82	366
8	Total conus defect type VSD	−3.9	85	559
9	Total conus defect type VSD	−6.9	105	453
10	Total conus defect type VSD	−2.7	138	396
11	Total conus defect type VSD	−0.9	152	348

LVEDV: left ventricle end-diastolic volume; PDA: patent ductus arteriosus; PV: pulmonary valve; VSD: ventricle septal defect; LVEDV%N: left ventricular end-diastolic volume (LVEDV) as a percentage of predicted normal; preop: preoperative; TAP: transannular patch

### Correlation between early echocardiographic parameters and preoperative LVEDVi or Nakata index

**Fig. 2:**
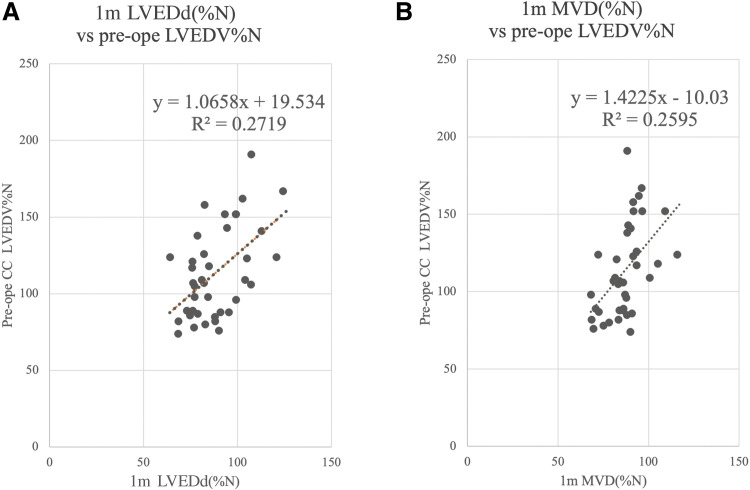
Correlations between preoperative LVEDV%N and echocardiographic measurements at 1 month of age. (**A**) Left ventricular end-diastolic dimension as a percentage of predicted normal (LVEDd%N); (**B**) effect of patent ductus arteriosus on the correlation; and (**C**) mitral valve diameter as a percentage of predicted normal (MVD%N). LVEDV%N: left ventricular end-diastolic volume as a percentage of predicted normal; LVEDd%N: left ventricular end-diastolic diameter as a percentage of predicted normal; MVD%N: mitral valve diameter % of predicted normal

**Fig. 3:**
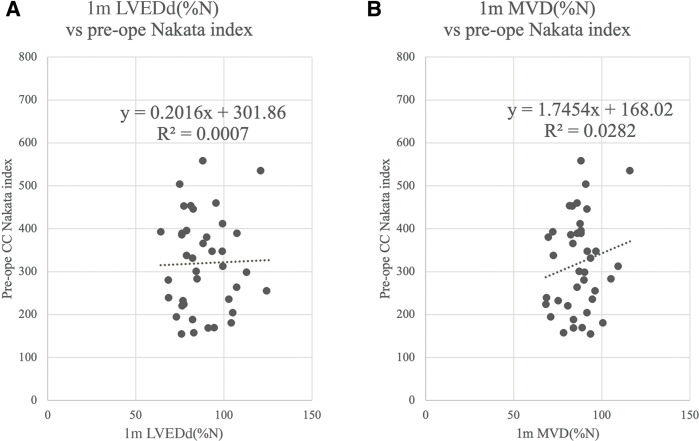
Correlations between Nakata index (pulmonary artery index) and echocardiographic measurements at 1 month of age. (**A**) Left ventricular end-diastolic dimension as a percentage of predicted normal (LVEDd%N) and (**B**) mitral valve diameter as a percentage of predicted normal (MVD%N). LVEDd%N: left ventricular end-diastolic diameter as a percentage of predicted normal; MVD%N: mitral valve diameter % of predicted normal

Preoperative LVEDV%N showed a weak but significant correlation with LVED diameter as a percentage of predicted normal (LVEDd%N) measured by TTE (R^2^ = 0.27, p <0.001) (**Fig. 2A**). PDA status did not influence the relationship between preoperative LVEDV%N and 1-month LVEDd%N (**Fig. 2B**). Similarly, a weak correlation was found between 1-month MVD as a percentage of predicted normal (MVD%N) and LVEDV%N prior to primary total correction (R^2^ = 0.26, p <0.001) (**Fig. 2C**). Nakata index was correlated with neither 1-month LVEDd%N nor MVD%N (**Fig. 3**).

Preoperative TTE and catheterization LVEDV data were available for comparison in 17 patients. TTE systematically underestimated LVEDV compared to catheterization (12.4 ± 4.9 vs. 17.2 ± 5.1 mL, p <0.001), with moderate correlation (r = 0.589, R^2^ = 0.347, p <0.05) but wide individual variation (TTE/catheter ratio, 0.72 ± 0.25; range, 0.44–1.41).

### Comparative analysis between LVEDV%N subgroups

Comparative analysis between patients with an LVEDV%N of 70%–80% (n = 4) and >80% (n = 37) who underwent primary repair revealed no significant differences in operative parameters: cardiopulmonary bypass time (202.0 ± 34.1 vs. 184.6 ± 47.8 minutes), aortic cross-clamp time (124.7 ± 24.8 vs. 116.2 ± 25.9 minutes), postoperative peak creatine kinase levels (278.7 ± 176.3 vs. 172.1 ± 65.7), or postoperative ventricular function assessed by discharge left ventricular ejection fraction (64.5% ± 4.9% vs. 69.1% ± 8.9%).

## Discussion

An old report by Graham et al. showed a case of a deceased boy whose postoperative LVEDVI was 26 mL/m^2^ (45% of LVEDV%N), with the main cause of death reported as low cardiac output due to poor left ventricular expansion.^3)^ Mortality following TOF repair may result from technical complications, such as left coronary injury or ventricular perforation during right ventricular outflow tract muscle resection, or from unexpected perioperative events. Consequently, the specific contribution of small left ventricular size to mortality rates remains uncertain.^14)^ However, Nomoto et al. reported that patients with a postoperative LVEDV%N of less than 70% tended to develop low cardiac output syndrome and required higher doses of dopamine postoperatively.^4)^ Similarly, Naito et al. recommended palliative shunt surgery for patients with an LVEDVI of 30 mL/m^2^ or less, as it posed a high risk of low cardiac output during the early perioperative period.^5)^ These findings, together with our observations, suggest that preoperative assessment of left ventricular size is essential before primary total correction in asymptomatic TOF patients.

The current AATS expert consensus recommends primary repair for asymptomatic TOF patients without specific mention of ventricular volume assessment criteria.^1)^ Our findings suggest that this generalized approach may benefit from individualized consideration, as 11.6% of asymptomatic patients had LVEDV%N ≤80%. While comparative analysis showed no significant differences in perioperative outcomes between LVEDV%N subgroups, the importance of preoperative volume assessment lies in identifying patients who may benefit from alternative surgical strategies, particularly considering implications for valve preservation and long-term cardiac function.

The use of single-plane LVEDV measurement represents both a limitation and a standardized approach in our study. While biplane angiography provides theoretically superior accuracy, single-plane methodology has been extensively validated in pediatric populations and remains clinically relevant when applied consistently. The clinical thresholds established by Naito et al. were derived using similar single-plane techniques, ensuring direct applicability to established clinical decision-making frameworks.

Our findings align with recent evidence supporting the potential for PV annular growth following systemic-to-pulmonary shunting. Nakashima et al. demonstrated significant pulmonary annulus enlargement after modified Blalock–Taussig shunt (Z-score improvement from −5.1 to −2.8, p = 0.0028), with PV preservation achieved in 64.7% of patients with mild-to-moderate pulmonary stenosis compared to 36.8% in the primary repair group.^9)^ This supports our observation in the patient with the smallest LVEDV%N (62%), where shunt placement resulted in substantial ventricular growth (120%) and successful valve-sparing repair.

The moderate correlation between preoperative TTE and catheterization LVEDV (r = 0.589), combined with systematic underestimation by TTE and wide individual variation, supports the continued clinical value of catheterization for precise ventricular volume assessment. This relationship suggests that echocardiography alone cannot reliably identify patients with critically diminished LVEDV who may benefit from alternative surgical strategies.

Since the end of the 20th century, limited institutions have reported excellent survival rates after neonatal TOF repair.^6,7)^ However, despite variations in patient severity, high mortality after neonatal repair compared to non-neonatal repair was documented by national database analysis.^8)^ Moreover, the extremely high rate of using TAP at neonatal repair may impair later right ventricular function. On the contrary, late-referral asymptomatic TOF patients tended to have small LVEDV due to long-standing reduced left ventricular preload, which has been reported to negatively affect post-primary repair mortality.^15)^ In our case, with the smallest LVEDV%N (62%), LVEDV%N increased to 120% after shunt placement, enabling subsequent valve-sparing repair. Previous studies have shown that palliative shunting promotes growth of both the PV annulus and branch PAs,^9–11)^ facilitating valve-sparing repair in patients with small PV annuli. In our series, TAP repair was required in all four patients with preoperative LVEDV%N between 70% and 80%. If these patients had been palliated by shunt, some patients may have avoided TAP repair. However, postoperative morbidity and mortality rates of the palliative shunt operations should not be neglected; thus, the risks of palliative shunt and the benefits of the valve-sparing repair for late right ventricular function must be weighed carefully. Considering these findings, we propose that primary TOF repair should be performed during the infantile period, after 3 months of age, with individual assessment of LV volume guiding the decision between primary repair and a staged approach.

Statistical analysis of TAP patients revealed no significant differences between those with diminished versus normal LVEDV regarding anatomical characteristics. This suggests that severe valvar and subvalvar stenosis, rather than ventricular underdevelopment, primarily determines the need for transannular repair. LVEDV assessment therefore provides complementary information for surgical planning beyond traditional anatomical evaluation.

The clinical challenge in our four patients with small LV volume and small PV annulus lies in balancing the known risks of palliative shunting against potential benefits of valve preservation. While these patients maintained SpO_2_ ≥85% and were clinically stable for primary repair, the universal requirement for TAP and variable postoperative outcomes raise questions about optimal surgical strategy. Recent evidence suggests that approximately two-thirds of patients with mild-to-moderate pulmonary stenosis can achieve valve preservation following a staged approach.^9)^ However, extending this concept to asymptomatic patients requires careful, individualized risk–benefit analysis. This assessment should consider institutional experience, family preferences, and long-term implications of both TAP-related pulmonary regurgitation and potential shunt-related complications.

This study has several main limitations. First, the relatively small sample size limits the strength of our conclusions. Second, we focused exclusively on asymptomatic TOF patients without symptomatic controls from the same period. While symptomatic patients also underwent catheterization (except in emergency cases), comparable LVEDV analysis was not feasible due to heterogeneous timing, varying degrees of symptoms, and different clinical presentations. Third, although cardiac magnetic resonance imaging is considered the gold standard for volumetric assessment, our measurements relied on monoplane cineangiography data due to institutional software constraints. Fourth, while the sample size of patients with an LVEDV%N of 70%–80% was small (n = 4), no substantial differences in perioperative parameters were observed compared to the remainder of the cohort. Fifth, the substantial time interval between initial echocardiography (median, 31 days) and preoperative catheterization (median, 4.7 months) limits the interpretation of TTE–catheter discrepancies. We did not perform analyses stratified by echo-catheter interval or examine near-concurrent measurements, precluding definitive assessment of whether observed discrepancies reflect primarily methodological differences or temporal ventricular growth patterns.

Despite these limitations, our findings demonstrate that not all TOF patients without a history of hypoxic spells or duct-dependent pulmonary circulation have LVEDV%N exceeding 60% before total correction. In our experience, patients with an LVEDV%N of less than 70% may benefit from a staged approach rather than primary repair. Notably, in the case with the smallest LVEDV%N (62%), LVEDV%N increased to 120% after shunt placement, enabling subsequent valve-sparing repair. These observations emphasize that left ventricular volume assessment before proceeding with primary repair remains essential in treatment strategy determination, even in asymptomatic TOF patients.

## Conclusion

Left ventricular volume assessment prior to primary repair is essential for optimal surgical strategy selection in asymptomatic TOF patients. While primary repair appears feasible in most cases, individualized evaluation identifies patients who may benefit from alternative approaches, including staged procedures that preserve PV function. Prognostic outcomes are excellent with either strategy, but volume-based assessment enables personalized decision-making that optimizes both immediate results and long-term cardiac function.

## Declarations

### Data availability

The data that support the findings of this study are available from the corresponding author, Akinori Hirano, upon reasonable request.
